# Immunological imprint on peripheral blood in kidney transplant recipients after two doses of SARS-CoV-2 mRNA vaccination in Japan

**DOI:** 10.3389/fmed.2022.999374

**Published:** 2022-09-28

**Authors:** Shinya Takiguchi, Yusuke Tomita, Saeko Uehara, Koichiro Tateishi, Norio Yamamoto, Michio Nakamura

**Affiliations:** ^1^Department of Transplant Surgery, Tokai University School of Medicine, Kanagawa, Japan; ^2^Department of Virology, Division of Host Defense Mechanism, School of Medicine, Tokai University, Kanagawa, Japan

**Keywords:** SARS-CoV-2 mRNA vaccination, kidney transplantation, cellular and humoral immune responses, immunological imprint, safety

## Abstract

The immunological imprint after two doses of severe acute respiratory syndrome–coronavirus 2 (SARS-CoV-2) mRNA vaccination for patients after kidney transplantation (KTx) remain unclear. This study included KTx recipients and volunteer healthy controls (HCs) who received two doses of SARS-CoV-2 mRNA vaccine (Pfizer BioNTech) from January 2021 to December 2021. We analyzed safety within 21 days after each vaccination dose and compared the immune response in peripheral blood mononuclear cells (PBMCs) between the two groups. No graft rejection was observed throughout this study. Adverse events were generally observed within 5 days. The KTx group exhibited a significantly lower degree of symptoms between doses 1 and 2 (*P* < 0.001). Increases in activated subsets of T and B cells expressing human leukocyte antigen (HLA)-DR and/or CD38 were observed in the HC group after dose 2 (both *P* < 0.001), with the greatest increases in HLA-DR^+^CD8^+^ T cells and CD38^+^CD19^+^ B cells (*P* = 0.042 and *P* = 0.031, respectively). In addition, PD1^+^CD8^+^ T cells—but not PD1^+^CD4^+^ T cells—increased significantly in the HC group (*P* = 0.027). In the KTx group, however, activated HLA-DR^+^, CD38^+^, and PD1^+^ cells remained at baseline levels. Immunoglobulin (Ig)G against SARS-CoV-2 was detected in only four KTx recipients (13.3%) after dose 2 (*P* < 0.001). Multivariate logistic regression analyses revealed that ΔHLA-DR^+^CD8^+^ T cells and ΔCD38^+^CD19^+^ B cells were significantly associated with IgG formation (both *P* = 0.02). SARS-CoV-2 mRNA vaccine generates impaired cellular and humoral immunity for KTx recipients. Results indicate the need for modified vaccination strategies in immunocompromised KTx recipients.

## Introduction

Severe acute respiratory syndrome–coronavirus 2 (SARS-CoV-2) is a respiratory virus of the beta-coronavirus family first described in December 2019 in China. Due to immunosuppressive therapy, solid organ transplant (SOT) recipients are immunocompromised and therefore at increased risk of developing severe coronavirus disease 2019 (COVID-19) infection (1–3). In Japan, the novel messenger RNA (mRNA)-based SARS-CoV-2 vaccines were approved in January 2021. While two doses of SARS-CoV-2 mRNA vaccination reportedly exhibit 95% efficacy, immunocompromised patients were not included in the large placebo-controlled trials of the vaccines (4, 5). Although several recent studies have examined the safety, local and systemic reactogenicity, and incidence of adverse events in SOT recipients after two doses of SARS-CoV-2 mRNA vaccination (6, 7), these studies lacked non-SOT controls. In our pre-study questionnaire, almost 60% of KTx recipients expressed worries over SARS-CoV-2 mRNA vaccination. Most of these concerns were related to safety, including anaphylactic and side reactions, interactions with immunosuppressive drugs, and graft rejection. Ou et al. reported that approximately 50% of SOT recipients refused vaccination for safety reasons (6).

COVID-19 infection has a variety of effects on host immunity. More than 10 weeks after COVID-19 infection, levels of cytotoxic CD3^+^CD8^+^ T cells co-expressing human leukocyte antigen (HLA)-DR and CD38 were found to be higher in peripheral blood compared with healthy control (HC) volunteers (8). In contrast, levels of CD25^+^Foxp3^+^CD4^+^ regulatory T cells (Tregs) were found to be lower in COVID-19 patients (8). Wang et al. showed that the function of T and B cells was associated with differences in severity of COVID-19 infection (9). Patients with extremely severe disease exhibited lower absolute numbers of CD3^+^CD4^+^ and CD8^+^ T cells and CD19^+^ B cells. In addition, the frequencies of IFN-*γ*–producing CD3^+^CD4^+^ and CD8^+^ T cells were dramatically increased in patients with extremely severe COVID-19. Thieme et al. reported no differences in the post–COVID-19 SARS-CoV-2–specific T cell response and expansion of effector-memory T cells between SOT recipients and non-immunosuppressed controls (10). However, there are no published reports of studies comparing the immunological imprint on peripheral blood in SOT recipients after two doses of SARS-CoV-2 mRNA vaccination. Changes in the cellular immune response in SOT recipients might increase the risk of *de novo* donor-specific anti–HLA antibody production, leading to graft rejection. Therefore, the aim of this study was to determine the safety and immunological imprint of two doses of SARS-CoV-2 mRNA vaccine (Pfizer BioNTech) on peripheral blood mononuclear cells (PBMCs) between immunocompromised kidney transplantation (KTx) recipients and HCs.

## Materials and methods

### Populations

Data were collected for all participants who provided written and oral informed consent. The study was approved by the Institutional Review Board of Tokai University Hospital at Isehara, Japan (#21-R007), and the study was conducted according to the principles of the Declaration of Helsinki. Participants received two intramuscular injections (0.3 mL each) of SARS-CoV-2 mRNA vaccine (Pfizer BioNTech) 21 days apart between January and December 2021. The renal function of all KTx recipients was assessed in our department approximately once each month. HCs, including KTx donors, were recruited through our outpatient care unit. A total of 128 participants recorded a safety sheet after SARS-CoV-2 mRNA vaccine injections: 78 KTx recipients and 50 HCs (16 KTx donors and 34 healthy volunteers). After participants were grouped based on characteristics, cellular and humoral immune responses in peripheral blood were evaluated after two vaccine doses in 12 HCs (8 KTx donors and 4 healthy volunteers) and 30 KTx recipients. No participants tested positive for SARS-CoV-2 infection over the duration of this study.

### Monitoring of post-vaccination safety and adverse events

A safety sheet for recording adverse event information was distributed to participants before the first vaccine dose. Participants self-reported specific local and systemic adverse events and use of any medications 1, 2, 3, 5, 7, 14, and 21 days after each vaccination dose. The following 11 adverse events were selected for monitoring based on previous randomized studies (4, 5): pain or redness and swelling at the injection site as local events, and fever, fatigue, headache, chills, vomiting, diarrhea, muscle pain, and joint pain as systemic events. Adverse events were quantified by scoring “Yes” responses as 1 and “No” responses as 0, and the sum of scores was recorded for days 1, 2, 3, 5, 7, 14, and 21 after each vaccination dose.

### Peripheral blood mononuclear cells and serum collection

Blood samples for isolation of PBMCs and serum were collected from 42 participants: 30 KTx recipients who had been followed-up for at least 6 months after KTx, and 12 HCs (8 KTx donors and 4 healthy volunteers). Blood samples were collected within 7 days before dose 1 (D1) of vaccination, within 7 days, and at 30 days after dose 2 (D2) of vaccination. PBMCs were isolated from 8 mL of venous blood by density gradient centrifugation using cell preparation tubes (BD Vacutainer^®^ CPTTM #362761; BD Biosciences, San Jose, CA). Isolated PBMCs and serum samples were stored in liquid nitrogen and a −80°C freezer, respectively.

### Flow cytometry

Flow cytometric assays were performed as described previously (11, 12). Briefly, PBMCs were thawed and co-cultured in a 24-well plate at 1.0 × 10^6^ cells/well in RPMI 1,640 with 10% fetal calf serum for 24 h at 37°C and 5% CO_2_. After overnight culture, the cells were labeled with the following anti-human monoclonal antibodies: CD3-APC Cy7 (SK7; BD Bioscience), CD4-Alexa700 (RPA-T4; BD Bioscience), CD8-BV480 (PRA-T8, BD Bioscience), CD19-BV421 (HIB19, Bio-Legend), HLA-DR-BV605 (G46-6, BD Bioscience), CD38-BV786 (HIT2, BD Bioscience), PD1-phycoerythrin (PE) (EH12.1, Bio-Legend), CD25-PECy7 (BC96; Bio-Legend), and IL7Ra (CD127)-BV650 (A019D5; Bio-Legend). Isotype controls, 7-AAD, and Fc blocker were included in all experiments. Multicolor flow cytometric analysis was performed using a BD FACS Fortessa analyzer (BD Biosciences) and FlowJo ver. 10 software (Tree Star Inc., Ashland, OR).

### Quantification of IgG antibodies specific for SARS-CoV-2

SARS-CoV-2 IgG II Quant assays were performed on an Abbott Alinity i platform following the manufacturer’s instructions (Abbott, USA). A serological assay based on the chemiluminescent immunoassay was used to determine the presence of IgG antibodies specific for SARS-CoV-2. Antibody tests were directed against the spike 1 protein receptor binding domain (S-RBD) at 30 days after D2 vaccination. The cut-off values were determined at ≥ 50 AU/mL.

### Statistical analysis

Data were analyzed using GraphPad Prism 9 (GraphPad Software, Inc., CA, USA). The significance of differences was evaluated using the chi-squared test for discrete variables and the Mann-Whitney *U* test for continuous variables. Factors affecting anti–S-RBD IgG formation were analyzed using univariate and multivariate logistic regression models. Statistical data for each cohort are expressed as mean ± SD. *P* < 0.05 was considered statistically significant in each comparative analysis (*P < 0.05, **P < 0.01, ***P < 0.001).

## Results

### Study population

The characteristics of KTx recipients and HCs are summarized in Table 1. Of the 50 HCs, 16 were KTx donors, and 34 were healthy volunteers. In our cohort, no significant differences were found between the groups in terms of age, sex, or renal function parameters. Most KTx recipients (92.3%) were administered three or more immunosuppressive drugs, including cyclosporine (CyA) or tacrolimus (Tac), mycophenolate mofetil (MMF), or mizoribine (MZ), everolimus (EVR), and a steroid. The time between KTx and D1 vaccination was 1846.7 ± 1644.1 days.

**TABLE 1:** Characteristics of KTx recipients and HCs who received two doses of SARS-CoV-2 mRNA vaccine (Pfizer BioNTech).

	KTx	HC	
	78	50	*P*
Age (years)	53.2 ± 11.7	51.9 ± 14.6	0.914
Sex (male/female)	40/38	21/29	0.305
sCr (mg/dL)	1.4 ± 0.7	1.1 ± 0.2	0.158
eGFR (mL/min/1.73 m^2^)	43.3 ± 12.2	45.2 ± 7.0	0.699
**Immunosuppression**		–	
Double	6		
Triple	41		
Quadruple	31		
TAC	67		
trough (ng/mL)	4.4 ± 1.1		
CyA	11		
trough (ng/mL)	67.9 ± 21.1		
MMF	75		
dose (mg)	888.1 ± 260.2		
MZ	1		
dose (mg)	300		
EVR	50		
trough (ng/mL)	4.2 ± 0.8		
Steroid	56		
dose (mg)	3.1 ± 1.3		
Desensitization therapy	44		
Duration between D1 and KTx (days)	1846.7 ± 1644.1	–	

mean ± SD, KTx, kidney transplantation; HC, healthy control; sCr, serum creatinine; eGFR, estimated glomerular filtration rate; TAC, tacrolimus; CyA, cyclosporine; MMF, mycophenolate mofetil; MZ, mizoribine; EVR, everolimus; D1, dose 1.

### Local and systemic reactogenicity

**FIGURE 1:**
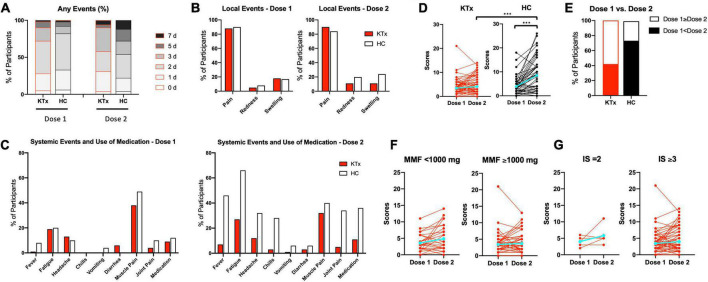
Local and systemic adverse reactions after each vaccination, as recorded by KTx recipients and HCs. **(A)** Comparison how many days KTx recipients and HCs suffered from any local or systemic adverse events after D1 and D2 vaccination. Day 0 is the day of vaccination. **(B,C)** Incidence of local **(B)** and systemic **(C)** adverse events. **(D)** Comparison of ‘Yes’ or ‘No’ scores and change in groups. **(E)** Percentage of scores D1 < D2 or D1 ≥ D2 in each group. **(F,G)** Comparison of scores and change with regard to MMF dose **(F)** and number of immunosuppressive drugs **(G)** in KTx recipients. Blue lines represent means. Data were analyzed using the Mann-Whitney *U* test (***P < 0.001).

No rejection was observed throughout this study. Almost all adverse events occurred within 7 days after vaccination (Figure 1A). Among HCs, post-D2 symptoms persisted longer than post-D1 symptoms (2.0 days after D1, 3.0 days after D2). Pain at the injection site as a local event was the most commonly reported adverse effect, and the rate was comparable between KTx recipients and HCs after D1 and D2 (88 vs. 90% after D1 and 90 vs. 84% after D2 in KTx recipients vs. HCs, respectively) (Figure 1B). Redness and swelling after D2 were reported more often by HCs than KTx recipients (11% vs. 20% after D1 and 11% vs. 24% after D2 in KTx recipients vs. HCs, respectively). HCs reported a markedly higher incidence of systemic events after D2 (Figure 1C). The most commonly reported systemic events among KTx recipients were fatigue and muscle pain (19 and 27% after D1, and 39% and 32% after D2, respectively), although these adverse events were more frequent in HCs after D1 and D2 (20 and 49% after D1, and 66 and 40% after D2, respectively). Only 1% of KTx recipients and 8% of HCs reported fever after D1, as compared with 7 and 46%, respectively, after D2.

HCs exhibited significantly higher adverse event scores, particularly after D2 (3.48 vs. 3.91 after D1 and 4.13 vs. 8.52 for KTx recipients vs. HCs, respectively; *P* < 0.001) (Figure 1D). Interestingly, adverse event scores decreased after D2 for approximately 40% of KTx recipients (Figure 1E). Based on a previous report (13), we performed sub-analyses of MMF dose and the number of immunosuppressive drugs for KTx recipients. KTx recipients who received < 1,000 mg of MMF and underwent double immunosuppressive therapy exhibited slightly increased adverse event scores after D2; however, no significant differences were found between the two groups (Figures 1F,G). No serious adverse events, anaphylaxis, or SARS-CoV-2 infection were reported in either the KTx recipient or HC groups throughout the study.

### SARS-CoV-2 mRNA vaccination did not affect peripheral blood CD3^+^ T cells and CD19^+^ B cells

**FIGURE 2:**
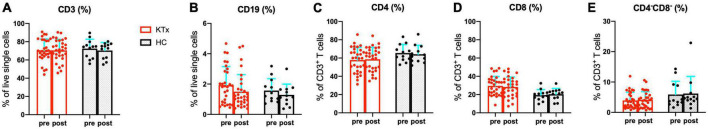
Quantification of major immune lymphocyte subsets among PBMCs. Comparison of the proportions of CD3^+^ T cells **(A)**, CD19^+^ B cells **(B)**, CD4^+^ T cells **(C)**, CD8^+^ T cells **(D)**, and CD4^–^CD8^–^ T cells **(E)** in KTx recipients (*n* = 30) and HCs (*n* = 12) pre- and post-vaccination. Bar plots depict mean ± SD. Data were analyzed using the Mann-Whitney *U* test.

Participants were grouped according to characteristics (Table 2), and the overall leukocyte populations in the peripheral blood were analyzed in 30 KTx recipients and 12 HCs after two doses of SARS-CoV-2 mRNA vaccination. Similar trends were observed in the proportions of CD3^+^ T cells, and CD19^+^ B cells pre- and post-vaccination in the HC and KTx groups (Figures 2A,B). Before vaccination (baseline), KTx recipients had a lower proportion of CD4^+^ T cells and a higher proportion of CD8^+^ T cells than HCs (Figures 2C,D). However, there were no significant differences between groups in the proportions of CD4^+^, CD8^+^, and CD 4^–^CD8^–^ T cells post-vaccination compared with baseline (Figures 2C–E).

**TABLE 2:** Characteristics of KTx recipients and HCs for which PBMCs were analyzed by flow cytometric assay.

	KTx	HC	
	30	12	*P*
Age (years)	54.4 ± 11.3	55.2 ± 15.3	0.842
Sex (male/female)	15/15	6/6	1
sCr (mg/dL)	1.2 ± 0.3	1.1 ± 0.2	0.652
eGFR (mL/min/1.73 m^2^)	45.2 ± 9.1	44.6 ± 6.0	0.951
**Immunosuppression**		–	
triple	13		
quadruple	17		
TAC	25		
CyA	5		
MMF	30		
EVR	25		
Steroid	12		

mean ± SD, KTx, kidney transplantation; HC, healthy control; sCr, serum creatinine; eGFR, estimated glomerular filtration rate; TAC, tacrolimus; CyA, cyclosporine; MMF, mycophenolate mofetil; EVR, everolimus.

### Activated CD3^+^ T cells increased markedly in HCs after SARS-CoV-2 mRNA vaccination

**FIGURE 3:**
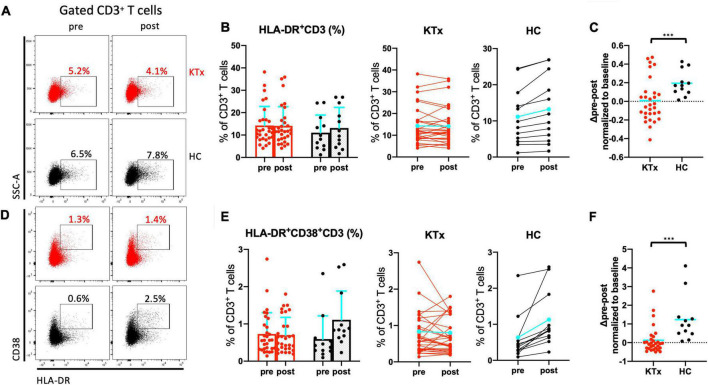
Activated CD3^+^ T cells increased after SARS-CoV-2 mRNA vaccination in HCs not but KTx recipients. Representative gating strategies and scatter plots of activated HLA-DR^+^ and/or CD38^+^ cells among CD3^+^ T cells in the two groups pre- and post-vaccination **(A,D)**. Comparison of the proportions and change in proportions of HLA-DR^+^ **(B)** and HLA-DR^+^CD38^+^ **(E)** CD3^+^ T cells in KTx recipients (left) and HCs (right) as measured by flow cytometry. Normalized increase in HLA-DR^+^ **(C)** and HLA-DR^+^CD38^+^ **(F)** CD3^+^ cells between pre- and post-vaccination. Bar plots depict mean ± SD. Blue lines and bars represent means. Data were analyzed using the Mann-Whitney *U* test (***P < 0.001).

We next assessed the post-vaccination expression of the activation markers HLA-DR and/or CD38 in CD3^+^ T cells (Figure 3). The proportions of the HLA-DR^+^CD3^+^ and HLA-DR^+^CD38^+^CD3^+^ activated T cell subsets were higher at baseline in KTx recipients than HCs (Figures 3A,B,D,E). After the second vaccination dose, HCs exhibited a higher proportion of HLA-DR^+^CD3^+^ T cells in the peripheral blood (Figure 3C). Remarkably, vaccinated HCs had a significantly higher proportion of CD3^+^ activated T cells co-expressing HLA-DR and CD38 (Figures 3D,E). The changes in HLA-DR^+^CD3^+^ and HLA-DR^+^CD38^+^CD3^+^ activated T cells normalized relative to baseline were dramatically greater in the HC group compared with the KTx group (both P < 0.001) (Figures 3C,F).

### HLA-DR^+^CD8^+^ and PD1^+^CD8^+^ T cells were induced by SARS-CoV-2 mRNA vaccination in HCs not but KTx recipients

**FIGURE 4:**
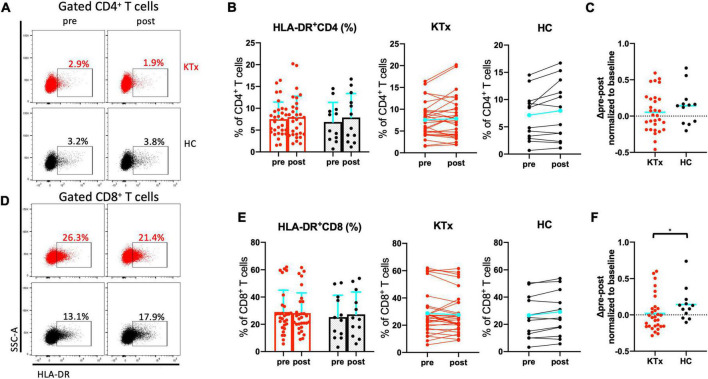
Activated CD8^+^ T cells were induced by SARS-CoV-2 mRNA vaccination in HCs. Representative gating strategies and scatter plots of activated HLA-DR^+^, PD1^+^, and Treg cells among T cell subsets in the two groups pre- and post-vaccination **(A,D)**. Comparison of the proportions and change in proportions of HLA-DR^+^ cells among CD4^+^ **(B)** and CD8^+^ **(E)** T cells in KTx recipients (left) and HCs (right) as measured by flow cytometry. Normalized increases in HLA-DR^+^ cells among CD4^+^ **(C)** and CD8^+^ **(F)** T cells between pre- and post-vaccination. Bar plots depict mean ± SD. Blue lines and bars represent means. Data were analyzed using the Mann-Whitney *U* test (*P < 0.05).

**FIGURE 5:**
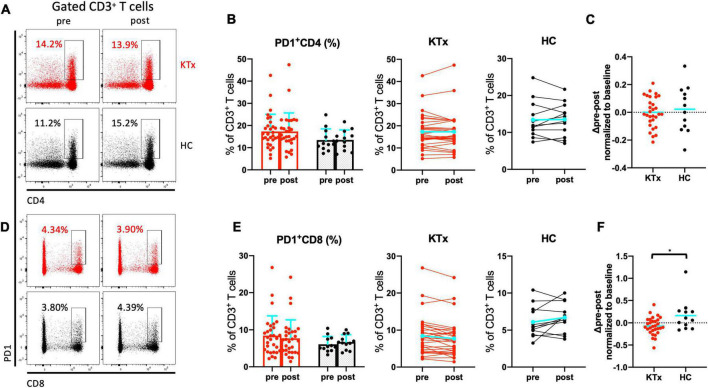
PD1^+^ CD8^+^ T cells were induced by SARS-CoV-2 mRNA vaccination in HCs. Representative gating strategies and scatter plots of activated PD1^+^ cells among T cell subsets in the two groups pre- and post-vaccination **(A,D)**. Comparison of the proportions and change in proportions of PD1^+^ cells among CD4^+^ **(B)** and CD8^+^ **(E)** T cells in KTx recipients (left) and HCs (right) as measured by flow cytometry. Normalized increases in PD1^+^ cells among CD4^+^ **(C)** and CD8^+^ **(F)** T cells between pre- and post-vaccination. Bar plots depict mean ± SD. Blue lines and bars represent means. Data were analyzed using the Mann-Whitney *U* test (*P < 0.05).

There was no absolute increase in the proportion of CD4^+^ T cells in either the KTx or HC group and no normalized increase in expression of the activation marker HLA-DR between baseline and post-vaccination (Figures 4A–C). In contrast, CD8^+^ T cells exhibited significantly increased HLA-DR expression in HCs after the second vaccination dose (Figures 4D–F). The HLA-DR^+^CD8^+^ T cell subset was exclusively associated with the difference in whole activated CD3^+^ T cells (P = 0.042), whereas there was no increase in activation of HLA-DR^+^CD4^+^ T cells in HCs even after receiving the second SARS-CoV-2 mRNA vaccination (*P* = 0.386). The proportion of cells expressing the acute T cell–exhaustion marker PD1 on CD8^+^ T cells also exhibited an increasing trend in HCs (Figures 5A,B,D,E). Furthermore, a significant increase in PD1^+^CD8^+^ T cells (but not PD1^+^CD4^+^ T cells) normalized relative to baseline was observed after D2 in the HC group compared with the KTx group (*P* = 0.027 and *P* = 0.710, respectively) (Figures 5C,F). Throughout vaccination, KTx recipients exhibited less-prominent T cell immune responses normalized relative to baseline as compared with HCs (Figures 3C,F, 4C,F, 5C,F). The proportion of CD25^+^CD127^–^CD4^+^ Tregs in KTx patients at baseline was significantly lower than that in HCs (Supplementary Figures 1A,B); however, there was no significant difference between groups in terms of the absolute increase in proportion and the normalized increase in these Tregs after vaccination (*P* = 0.162) (Supplementary Figures 1B,C).

### Activated CD38^+^CD19^+^ B cells and anti–S-RBD IgG antibody formation increased dramatically in HCs after SARS-CoV-2 mRNA vaccination

**FIGURE 6:**
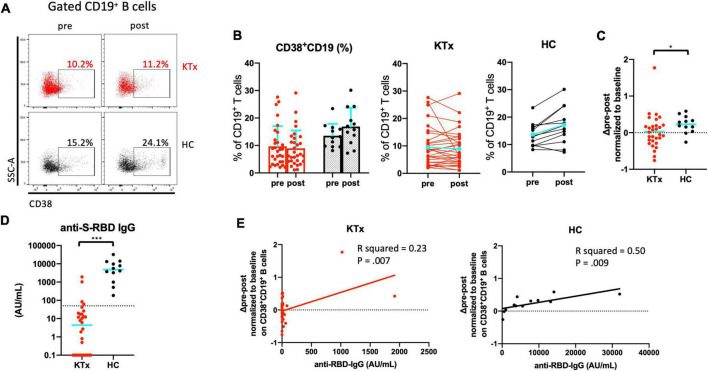
Activated CD19^+^ B cells increased after SARS-CoV-2 mRNA vaccination in HCs. **(A)** Representative gating strategies and scatter plots of activated CD38^+^ cells among CD19^+^ B cells in the two groups pre- and post-vaccination. **(B)** Comparison of the proportion and change in proportion of CD38^+^CD19^+^ B cells in KTx recipients (left) and HCs (right) as measured by flow cytometry. **(C)** Normalized increase in CD38^+^CD19^+^ B cells between pre- and post-vaccination. **(D)** Serologic S-RBD IgG antibody response against SARS-CoV-2 at 30 days after D2 vaccination. **(E)** *Scatter plot of anti-S-RBD IgG values and normalized increase in CD38^+^CD19^+^ B cells by flow cytometric analysis. Data were obtained from 42 samples (30 in KTx group and 12 on HC group), and a moderate correlation was found in HC group.* Bar plots depict mean ± SD. Blue lines and bars represent means. Data were analyzed using the Mann-Whitney *U* test (*P < 0.05, ***P < 0.001).

We analyzed expression of the activation marker CD38 on CD19^+^ B lymphocytes after vaccination (Figure 6). We confirmed that KTx recipients and HCs had similar baseline proportions of CD38^+^CD19^+^ B cells among PBMCs (Figures 6A,B). Although there was no difference after vaccination in the proportion of CD38^+^CD19^+^ B cells in KTx recipients, that in HCs was significantly increased after the second dose (Figures 6B,C). In the HC group only, a significant difference was also observed in the change in the proportion of activated CD38^+^CD19^+^ B cells normalized relative to baseline (*P* = 0.031) (Figure 6C). Nevertheless, all HCs exhibited an RBD-IgG antibody response at 30 days after D2, and KTx recipients showed a significant lower antibody response against SARS-CoV-2 (*P* < 0.001) (Figure 6D). *According to the simple liner regression analysis, the normalized increase in CD38^+^CD19^+^ B cells moderately correlated with anti-RBD-IgG antibody values in HC groups (R squared = 0.50, P = 0.009), not but in KTx groups (R squared = 0.23, P = 0.007) (Figure 6E).*

### ΔActivated HLA-DR^+^CD8^+^ T cells and Δactivated CD38^+^CD19^+^ B cells significantly impacted detection of anti–S-RBD IgG against SARS-CoV-2

Finally, we analyzed factors related to the formation of anti–S-RBD SARS-CoV-2 IgG using univariate and multivariate logistic regression models (Table 3). Univariate regression analyses showed that ΔHLA-DR^+^CD8^+^ T cells, ΔPD1^+^CD8^+^ T cells, and ΔCD38^+^CD19^+^ B cells were significantly associated with anti–S-RBD IgG formation. Multivariate logistic regression analyses showed that ΔHLA-DR^+^CD8^+^ T cells and ΔCD38^+^CD19^+^ B cells were significantly associated with anti–S-RBD SARS-CoV-2 IgG formation (*P* = 0.02, odds ratio = 6.60 and *P* = 0.02, odds ratio = 7.17, respectively).

**TABLE 3:** Factors related to anti–S-RBD IgG formation estimated using univariate and multivariate logistic regression analyses (*n* = 42).

		Univariate analysis			Multivariate analysis	
	OR	95% CI	*P*	OR	95% CI	*P*
Δ HLA-DR^+^CD8^+^ T cells	4.8	1,28–21.2	0.02	6.6	1.35–43.1	0.02
Δ PD1^+^CD8^+^ T cells	4.15	1.14–16.9	0.03	2.18	0.43–10.6	0.32
Δ CD38^+^CD19^+^ B cells	5.06	1.26–26.1	0.03	7.17	1.41–52.1	0.02

OR, odds ratio; CI, confidence intervals; S-RBD, spike 1 protein receptor binding domain.

## Discussion

As immunocompromised patients such as KTx recipients are considered at high risk of severe COVID-19 (14, 15), SOT patients were excluded from the clinical trials of the SARS-CoV-2 mRNA vaccines (4, 5). Markedly lower immunization after COVID-19 vaccination in SOT recipients was recently reported (13, 16–19). *Sanders et al. showed that the seropositivity rates were persisted for up to 6 months after D2; however, anti-RBD-IgG antibody values and IFN-*γ* production by T cell responses were dramatically decreased in KTx recipients (19)*. Therefore, evaluation of the cellular and humoral immune responses in the peripheral blood of KTx recipients after two doses of SARS-CoV-2 mRNA vaccine is urgently needed. We thus performed a healthy volunteer–controlled cohort study of immunocompromised KTx recipients receiving two doses of SARS-CoV-2 mRNA vaccine (Pfizer BioNTech). No kidney graft rejection was observed in this study. Our results showed that the SARS-CoV-2 mRNA vaccine was safe for KTx recipients and generated lower local and systemic reactogenicity and much lower cellular and humoral immune responses in the peripheral blood compared with HCs. No serious adverse events, anaphylaxis, or SARS-CoV-2 infection were noted in either group throughout the study.

Our findings regarding local and systemic reactogenicity were comparable to those of large clinical trials of the novel SARS-CoV-2 mRNA vaccine in HCs (4, 5). In the KTx group, pain at the injection site, fatigue, and muscle pain were the most commonly reported adverse event symptoms, and the incidence of systemic reactogenicity was <50%. This trend was consistent with previous reports (6, 7). With the exceptions of pain at the injection site and muscle pain, adverse reactions in both groups were mild and less common after the first vaccination dose. In HCs, systemic reactogenicity after D2 was severe and more common than that after D1, however. In contrast, the level of reactogenicity in the KTx group was similar after both vaccination doses, and the incidence of fever was much lower after D2. Therefore, the KTx group exhibited a significantly lower degree of adverse event symptoms between doses 1 and 2. Almost all adverse events following doses 1 and 2 generally resolved within 7 days. Taken together, these results indicate that the SARS-CoV-2 mRNA vaccine is safe for not only healthy individuals but also immunocompromised KTx recipients.

Previous studies of patients suffering from COVID-19 infection have reported a decrease in the number of CD3^+^ T cells with increasing disease severity. Additionally, the hyperfunction of CD4^+^ and CD8^+^ T cells is correlated with the pathogenesis of severe infection (9, 20). In particular, cytotoxic CD8^+^ T cells, not but CD4^+^ T cells, are activated and exhibit increased HLA-DR and/or CD38 expression (8, 9). Expression of HLA-DR on T cells is correlated with upregulated T cell activation (21). Moreover, CD38 surface expression has been shown to enhance inflammatory cytokine production, which coincides with CD3^+^ T cell activation (22). Although the post-vaccination proportions of activated HLA-DR^+^CD4^+^ T cells were comparable between the two groups, the majority of activated HLA-DR^+^CD38^+^CD3^+^ T cells observed in this study in the HC group were CD8^+^ T cells. Thus, peripheral blood activated CD8^+^ T cells appear to be the most important immune cells in the initial response in the early stage of SARS-CoV-2 mRNA vaccination, as well as in the response to COVID-19 infection.

PD1^+^ T cells serve as a marker of exhausted T cells. T cell exhaustion has been reported with viral infections, malignancies, transplantation, and situations associated with persistent inflammation (23–25). Whether PD1^+^ T cells are upregulated in the response to acute COVID-19 infection or SARS-CoV-2 mRNA vaccination remains unclear, however. Wang et al. reported that the frequency of PD1 and Tim3 expression on CD8^+^ T cells is increased in patients with extremely severe COVID-19 infection (9). Consistent with this result, PD1^+^CD8^+^ T cells were induced by SARS-CoV-2 mRNA vaccination in HCs in the present study. When comparing the exhaustion and expression of T cells after two doses of the vaccine, both groups exhibited differences between CD4^+^ and CD8^+^ T cells. Compared with KTx recipients, the expression of exhaustion markers such as PD1 on CD8^+^ T cells was elevated in HCs after vaccination.

For mRNA vaccines against influenza viruses, vaccine-induced B cell responses in CD19^+^ B cell compartments reportedly persist for 14 days after booster vaccination (26). *Recently, Schuller et al. reported that transitional B cells in total CD19^+^ B cell compartment were associated with antibody production against SARS-CoV-2 (27).* We also found that the proportion of activated CD38^+^CD19^+^ B cells increased markedly in HCs within 7 days after D2 of SARS-CoV-2 vaccination. These B cell findings might be associated with the production of SARS-CoV-2–specific S-RBD IgG antibodies.

In KTx recipients, impaired cellular and humoral immunity in the peripheral blood after SARS-CoV-2 vaccination was observed throughout this study. The most remarkable difference between the KTx and HC groups was that KTx recipients were treated with standard immunosuppressive therapy to maintain renal function after transplantation. To prevent *de novo* donor-specific anti–HLA antibody production and graft rejection, most of these patients were treated with a protocol involving multiple immunosuppressive agents, including CyA or Tac, MMF or MZ, or EVR and a steroid (28). A recent study reported that *in vitro* SARS-CoV-2 spike-specific CD8^+^ T cell responses were significantly reduced in cells from KTx recipients compared with cells from HCs (29). In addition, Rincon-Arevalo et al. reported that the incidence of RBD-specific circulating B cells and plasmablasts was correlated with immunoglobulin responses in their study; however, KTx recipients did not develop an IgG response (30). In liver transplantation, Rabinowich et al. reported that lower renal function and triple-therapy immunosuppression, particularly including MMF, were significant risk factors for a lower humoral immune response (13). Consistent with these reports, we showed that KTx recipients who received two doses of SARS-CoV-2 mRNA vaccine exhibited impaired cellular and humoral immunity. Taken together, these results strongly suggest that vaccination protocols for immunocompromised patients need to be revised.

This study has several limitations. First, the sample size of our cohort was relatively small and involved only a single center. Second, we only analyzed unstimulated PBMCs to assess the cellular and humoral immune responses after SARS-CoV-2 mRNA vaccination. A further analysis of SARS-CoV-2–specific PBMCs *in vitro* is needed in order to evaluate some of the well-known factors that affect immunocompromised patients. Third, we did not perform any biopsies or *de novo* donor-specific antibody testing after vaccination. However, no serious rejection was observed throughout this study. A strength of this study is that we assessed safety and cellular immune responses after two doses of SARS-CoV-2 mRNA vaccination in KTx recipients in comparison with HCs including KTx donors. This is the first study to show the significance of immunological imprint, especially in Asian immunocompromised KTx recipients after vaccination with the Pfizer BioNTech product. In addition, our data suggest that activated cells, particularly the HLA-DR^+^CD8^+^ and CD38^+^CD19^+^ B cell subsets, play a key role in the response to SARS-CoV-2 mRNA vaccination.

In summary, we demonstrated that the SARS-CoV-2 mRNA vaccine (Pfizer BioNTech) is safe for KTx recipients and generates lower cellular and humoral immune responses in KTx recipients compared with HCs. These data might help address the benefits and the need for modified vaccination strategies in immunocompromised KTx recipients.

## Data availability statement

The raw data supporting the conclusions of this article will be made available by the authors, without undue reservation.

## Ethics statement

The studies involving human participants were reviewed and approved by the Institutional Review Board of Tokai University Hospital (Isehara, Japan). The patients/participants provided their written informed consent to participate in this study.

## Author contributions

YT and MN designed the research and study. ST, YT, SU, and KT performed the research and study. YT, KT, and NY contributed the important reagents. ST and YT participated in the data collection, analysis, and writing of the manuscript. YT, NY, and MN participated in the revising of the manuscript. All authors contributed to the article and approved the submitted version.

## Supplementary material

Supplementary Figure 1 CD25^+^CD127^–^CD4^+^ Treg cells were not associated with SARS-CoV-2 mRNA vaccination. **(A)** Representative gating strategies and scatter plots of Treg cells among T cell subsets in the two groups pre- and post-vaccination. **(B)** Comparison of the proportion and change in proportion of Treg cells in KTx recipients (left) and HCs (right) as measured by flow cytometry. **(C)** Normalized increase in Treg cells between pre- and post-vaccination. Bar plots depict mean ± SD. Blue lines and bars represent means. Data were analyzed using the Mann-Whitney *U* test.

## Abbreviations

Treg: regulatory T cell
KTx: kidney transplantation
HC: healthy control
PBMC: peripheral blood mononuclear cell
SOT: solid organ transplantation
SARS-CoV-2: severe acute respiratory syndrome–coronavirus 2
COVID-19: coronavirus disease 2019
mRNA: messenger RNA
HLA: human leukocyte antigen
D1: dose 1
D2: dose 2
PE: phycoerythrin
CyA: cyclosporine
Tac: tacrolimus
MMF: mycophenolate mofetil
MZ: mizoribine
EVR: everolimus
sCr: serum creatinine
eGFR: estimated glomerular filtration rate
Ig: immunoglobulin
S-RBD: spike 1 protein receptor binding domain.
