# Supplementary figures and images for: Immunological imprint on peripheral blood in kidney transplant recipients after two doses of SARS-CoV-2 mRNA vaccination in Japan
Source: Front Med (Lausanne). 2022 Sep 28;9:999374. doi: 10.3389/fmed.2022.999374 (PMC9553995; doi:10.3389/fmed.2022.999374)

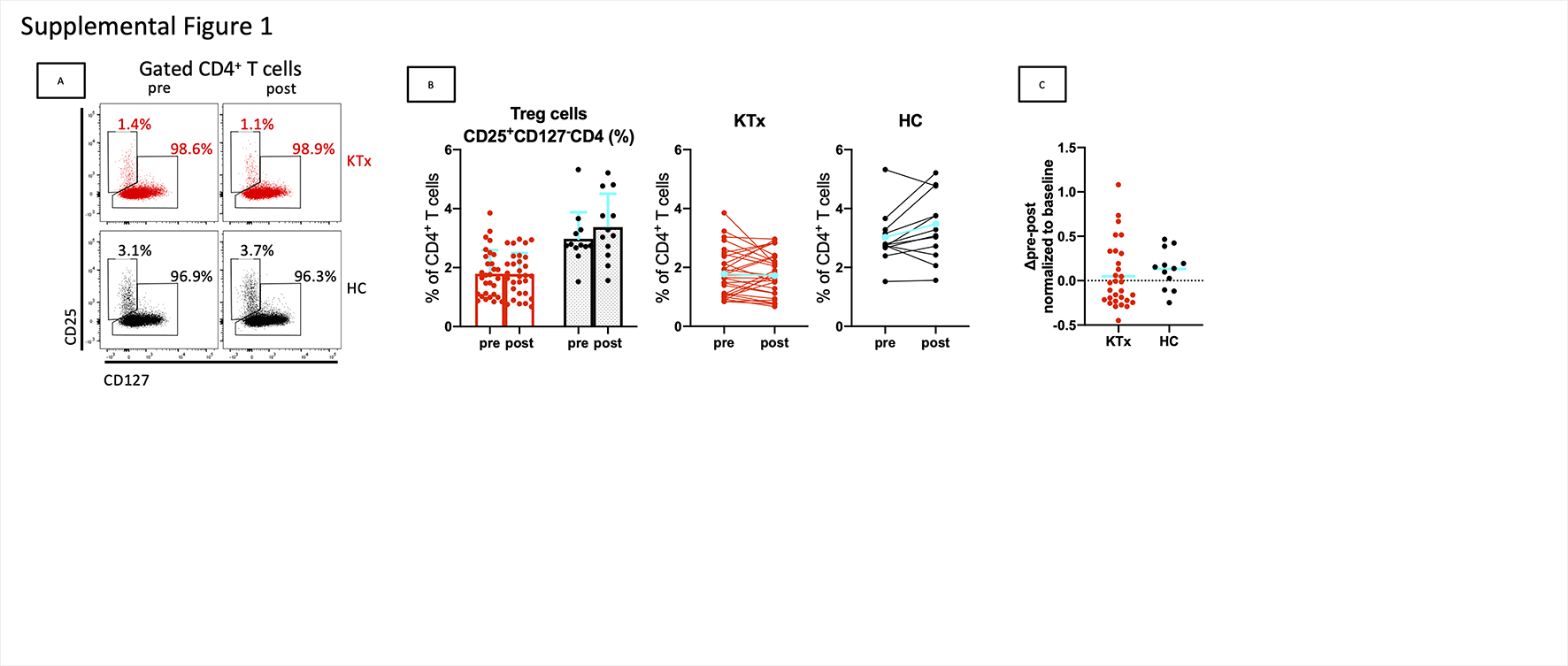

Supplement: Supplementary Figure 1 — CD25+CD127–CD4+ Treg cells were not associated with SARS-CoV-2 mRNA vaccination. (A) Representative gating strategies and scatter plots of Treg cells among T cell subsets in the two groups pre- and post-vaccination. (B) Comparison of the proportion and change in proportion of Treg cells in KTx recipients (left) and HCs (right) as measured by flow cytometry. (C) Normalized increase in Treg cells between pre- and post-vaccination. Bar plots depict mean ± SD. Blue lines and bars represent means. Data were analyzed using the Mann-Whitney U test. [file Image_1.TIFF]
